# Shark depredation: future directions in research and management

**DOI:** 10.1007/s11160-022-09732-9

**Published:** 2022-11-15

**Authors:** J. D. Mitchell, J. M. Drymon, J. Vardon, P. G. Coulson, C. A. Simpfendorfer, S. B. Scyphers, S. M. Kajiura, K. Hoel, S. Williams, K. L. Ryan, A. Barnett, M. R. Heupel, A. Chin, M. Navarro, T. Langlois, M. J. Ajemian, E. Gilman, E. Prasky, G. Jackson

**Affiliations:** 1 Queensland Government, Department of Agriculture and Fisheries, Ecosciences Precinct, 41 Boggo Road, Dutton Park, QLD 4102 Australia; 2 Mississippi State University, Coastal Research and Extension Center, 1815 Popps Ferry Road, Biloxi, MS 39532 USA; 3 Mississippi-Alabama Sea Grant Consortium, 703 East Beach Drive, Ocean Springs, MS 39564 USA; 4 Southern Cross University, Lismore, NSW Australia; 5 Department of Primary Industries and Regional Development, Western Australian Fisheries and Marine Research Laboratories, 39 Northside Drive, Hillarys, WA 6025 Australia; 6 Institute for Marine and Antarctic Studies, University of Tasmania, 20 Castray Esplanade, Battery Point, TAS 7004 Australia; 7 Coastal Sustainability Institute, Department of Marine and Environmental Sciences, Northeastern University, Nahant, MA 01908 USA; 8 Social Science Environmental Health Research Institute, Northeastern University, Boston, MA 02115 USA; 9 Department of Biological Sciences, Florida Atlantic University, 777 Glades Road, Boca Raton, FL 33431 USA; 10 Centre for Sustainable Tropical Fisheries and Aquaculture, James Cook University, Bldg 34 James Cook Drive, Douglas, QLD 4811 Australia; 11 School of Biological Sciences, The University of Queensland, St Lucia, Qld 4072 Australia; 12 Biopixel Oceans Foundation, Cairns, QLD Australia; 13 Marine Data Technology Hub, James Cook University, Townsville, QLD 4811 Australia; 14 School of Biological Sciences, The University of Western Australia, Crawley, WA Australia; 15 The Oceans Institute, University of Western Australia, Crawley, WA Australia; 16 Harbor Branch Oceanographic Institute, Florida Atlantic University, 5600 US 1 North, Fort Pierce, FL 34946 USA; 17 Pelagic Ecosystems Research Group, Honolulu, HI USA; 18 Heriot-Watt University, Edinburgh, UK

**Keywords:** Human-wildlife conflict, Fisheries management, Social-ecological systems, Shark behaviour

## Abstract

Shark depredation is a complex social-ecological issue that affects a range of fisheries worldwide. Increasing concern about the impacts of shark depredation, and how it intersects with the broader context of fisheries management, has driven recent research in this area, especially in Australia and the United States. This review synthesises these recent advances and provides strategic guidance for researchers aiming to characterise the occurrence of depredation, identify the shark species responsible, and test deterrent and management approaches to reduce its impacts. Specifically, the review covers the application of social science approaches, as well as advances in video camera and genetic methods for identifying depredating species. The practicalities and considerations for testing magnetic, electrical, and acoustic deterrent devices are discussed in light of recent research. Key concepts for the management of shark depredation are reviewed, with recommendations made to guide future research and policy development. Specific management responses to address shark depredation are lacking, and this review emphasizes that a “silver bullet” approach for mitigating depredation does not yet exist. Rather, future efforts to manage shark depredation must rely on a diverse range of integrated approaches involving those in the fishery (fishers, scientists and fishery managers), social scientists, educators, and other stakeholders.

## Introduction

Depredation, where a predator (e.g. a shark, cetacean, pinniped, seabird, squid, large teleost) completely or partially consumes an animal caught by fishing gear, is an issue in many fisheries around the world and in recent years has received increasing attention from researchers and fishery managers (Gilman et al. 2007; IOTC 2007; Mitchell et al. 2018a; Tixier et al. 2021). Shark depredation, in particular, has become a focal issue in a range of commercial, small-scale and recreational fishing contexts (Mitchell et al. 2018a). Depredation is a form of human-wildlife conflict (HWC) that has become a highly topical and emotive subject in many regions and generates polarising views due to its intersection with the broader context of fisheries management issues, such as declining fish stocks (Britten et al. 2021), increased recreational fishing participation (Arlinghaus et al. 2021) and the global push towards conserving historically over-harvested and potentially now recovering shark populations (Carlson et al. 2019; Pacoureau et al. 2021). There are a range of negative biological, economic, and social impacts from shark depredation, including, but not limited to, increased mortality of target species, loss or damage of catch and fishing gear and associated revenue, damage to the fishing experience (especially for recreational fishers), increasingly hostile views towards sharks, and retaliatory killing of sharks. Furthermore, higher shark depredation rates can lead to higher catchability risk and concomitant bycatch fishing mortality in some fisheries where the fishing gear used is capable of catching sharks (e.g. longlines, droplines), which is a concern due to the poor conservation status of many shark populations (Dulvy et al. 2014; Pacoureau et al. 2021) and because high shark catch rates reduce operational efficiency in fisheries where sharks are not retained.

Research into shark depredation dates back to the 1950s and remained at low levels until 2000, after which there was a notable increase in published literature (Gilman et al. 2007; Mitchell et al. 2018a). This likely reflects growing awareness of the issue amongst fisheries scientists and increasing calls from stakeholders to address its occurrence and impacts. Much of the early research focused on quantifying depredation rates in commercial longline fisheries (Sivasubramaniam 1964; Hirayama 1976; Mandelman et al. 2008; MacNeil et al. 2009). More recently, the focus has shifted towards recreational fisheries, particularly in Australia and the United States, where there are large recreational fishing communities that have become increasingly vocal about the need to mitigate shark depredation. This may be driven in part by increasing attention given to depredation in regular media (Major 2020; van Hoose 2021) and social media platforms (e.g. Sportsmen Fighting for Marine Balance Facebook page). As a result, there has been a strong focus on investigating shark depredation in recreational, charter (larger ‘for-hire’ vessels with guides, which typically carry > 10 fishers) and commercial fisheries in these countries, with recent studies quantifying depredation rates (Mitchell et al. 2018a; Ryan et al. 2019; Carmody et al. 2021), identifying shark species involved (Drymon et al. 2019; Fotedar et al. 2019; Mitchell et al. 2019; Vardon et al. 2021) and investigating changes in shark behaviour in the context of depredation (Mitchell et al. 2020, 2021). However, studies exploring the human dimensions of depredation conflicts remain relatively scarce.

**Fig. 1:**
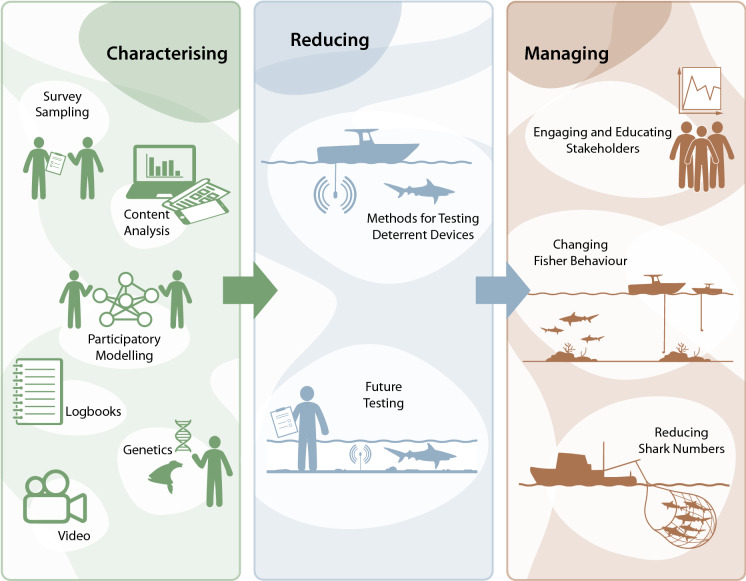
The multi-faceted aspects of characterising, reducing and managing depredation

Due to this recent increase in research on depredation and an increasingly strong stakeholder focus on the issue across all fishing sectors, there is a need to review progress to date and identify priority areas for future research. This review will synthesise recent advances in the field of shark depredation research, describe best-practice methods to characterise both the social and biological aspects of depredation, and highlight key areas for future targeted research, framed within the context of how fisheries managers and fishers can apply research to characterise, reduce and manage shark depredation (Fig. 1).

## Characterising shark depredation

Depredation occurs within complex systems that include multiple elements across the social and biological sciences. A comprehensive characterisation of the myriad social and biological aspects of depredation is a critical precursor to effectively reducing and managing this human-wildlife conflict.

### Social science

Research on the human dimensions of depredation is only just beginning. This review outlines findings of the few studies conducted to date and describes the methods and approaches that could be applied in future research. Research on HWCs in terrestrial environments has revealed that merely understanding the physical conflict between humans and wildlife only addresses a fraction of the problem. Often, HWCs are less driven by the direct conflicts with wildlife (Fraser-Celin et al. 2018). Instead, they stem from differences in human values, beliefs, or attitudes, such as balancing conservation goals with community well-being (e.g. Bagchi and Mishra 2006; Simpfendorfer et al. 2021), which must be identified and understood to adequately address and effectively resolve contentious HWCs (Guerra 2019). Despite the increased frequency of fisher–shark conflicts, shark depredation research has only recently started to draw on such insights from terrestrial HWC studies (Tixier et al. 2021). Yet, understanding what drives human beliefs has helped navigate discord between negative human–shark interactions (e.g. shark bites) and shark conservation efforts (Pepin-Neff and Wynter 2017; Niella et al. 2021), and may prove useful in better navigating the shark depredation conflict. To date, several social science frameworks have been used to explore the conflict (Table 1).

**Table 1:** Study design elements from published studies on fishers’ attitudes to shark encounters and occurrence of shark depredation while fishing

Study element	Study							
	Gilman et al. (2008)	Drymon and Scyphers (2017)	Mitchell et al. (2018b)	Ryan et al. (2019)	Carmody et al. (2021)	Iwane et al. (2021	Casselberry et al. (2022	Coulson et al. (2022
Sampling design	Face to face interviews at ports	Online questionnaires	Face to face interviews at boat ramps	Computer Assisted Telephone Interviews	Commercial fishing daily logbooks	Semi-structured interviews and participant observations	Online questionnaires	Computer Assisted Telephone Interviews and web-based (online) questionnaires
Sampling frame	Vessel captains, fishing masters, crew, vessel owners, cooperative staff and port officials	Recreational saltwater fishing licence holders (Florida residents, 18 years and above)	Recreational fishers launching vessels from four public boat ramps in study region	Commercial and charter fishers, recreational boat fishing licence holders (18 years and above)	Commercial vessels (with more than 10 vessel days over 13 years of reporting)	Recreational small boat fishers and community-based shark-tagging project	Recreational saltwater anglers (North Amercian residents, 18 years and above)	Charter fishers, Recreational boat fishing licence holders (18 years and above)
Primary Sampling Unit	Person	Person	Sample day (n = 40 survey days)	Person	Vessels (n = 32)	Person	Person	Person
Number of interviews	149 fishers (including associated fishing staff)	521 fishers	403 fishers	906 fishers	13,616 fishing sessions	29 fishers	541 fishers	1340 fishers
Sample selection	Snowball sampling	Random sampling	Systematic random sampling	Random sampling	Census	Snowball sampling; self-selection	Snowball sampling	Random sampling; self-selection
Stratification	8 countries, 12 fisheries, 24 seaports	None	Season, day type (weekday/weekend)	Residential region	3 fishery zones	None	None	None
Study period	January–December 2006	August–September 2013	July 2015–May 2016	August–November 2016	2006–2018	September 2017–June 2018	July 2019–January 2020	March–April 2020
Study location	Australia, Chile, Fiji, Italy, Japan, Peru, South Africa, USA	USA (Florida)	Oceania (Western Australia)	Oceania (Western Australia)	Oceania (north Western Australia)	USA (Hawai‘i)	USA	Oceania (Western Australia)
Key objectives	Understand shark catch and depredation rates; identify attitudes, behaviours, incentives and practices for avoidance/mitgation	Understand attitudes and perceptions towards shark conservation and sustainability	Quantify occurrence of depredation (spatial variation and frequency) and drivers of depredation	Quantify occurrence of depredation (fishing methods and geographic areas) and attitudes to depredation	Quantify depredation rates and drivers (fishing activity and environmental variables)	Explore perceptions of conflicts in fisher–shark interactions and solutions to address conflicts	Understand extent (target species), behavioural response and fishers' perceptions of depredation	Understand fisher behaviour associated with depredation and mitigation methods
Fishing sector	Commercial (small-scale artisanal and large-scale longline fisheries)	Recreational	Recreational	Commercial, charter and recreational	Commercial (Mackerel fishery)	Commercial, charter and recreational	Recreational	Charter and recreational

#### Survey sampling

Survey sampling utilising questionnaires (where data are self-reported by the respondent) or interviews (both structured and semi-structured; where data are recorded by an interviewer) represent the most popular social science strategies to understand shark depredation impacts, including rates of depredation and fishers’ attitudes, perceptions, and behaviours (Gilman et al. 2008; Drymon and Scyphers 2017; Ryan et al. 2019; Mitchell et al. 2018a, b; Casselberry et al. 2022). Survey designs aim to maximise survey participation, using a formal list of structured questions relevant to the survey objectives and appropriate for the contact method. Published studies on fisher–shark interactions have included online questionnaires (e.g. Casselberry et al. 2022), computer assisted telephone interviews (e.g. Ryan et al. 2019), and face-to-face interviews at boat ramps (e.g. Mitchell et al. 2018b) (Table 1). Such approaches can quantify physical phenomena or responses associated with depredation, as well as illuminating less tangible aspects (e.g. attitudes). For example, a state-wide survey on shark depredation across commercial, charter and recreational fishing sectors in Western Australia identified regional depredation hotspots and characterised fishers’ concerns around the issue (Ryan et al. 2019). Additionally, boat ramp surveys of recreational fishers in Western Australia quantified depredation frequency and identified potential drivers of these interactions (Mitchell et al. 2018b). Surveys have been useful to document fishers’ mitigation strategies (Coulson et al. 2022) and understand behavioural responses of fishers to depredation (Casselberry et al. 2022). Though not directly targeting depredation, Drymon and Scyphers (2017) also used online surveys to gauge fisher attitudes, perceptions, and beliefs about shark conservation, finding fishers often view sharks as competitors. Survey sampling approaches can help guide and prioritise future depredation research. However, despite being a useful tool, surveys can have limitations, particularly with respect to minimising survey errors under potentially limited resourcing and lack the ability to explore nuances or new ideas beyond their initial design.

Though less widely applied in shark depredation research, semi-structured interviews, which use open-ended questions to prompt discussion that allows the interviewer to explore responses, are a useful tool to characterise the respondent’s attitudes, behaviours and knowledge, as well as gaining additional insights into their beliefs and values with fewer constraints (Newing et al. 2010). While semi-structured interviews are longer and more in-depth than structured questionnaires and interviews, they can uncover less obvious conflicts, values, perceptions, and opinions surrounding depredation. Semi-structured interviews can be conducted through targeted focus groups to facilitate ongoing questions and review of emerging trends and engage fishers in an ongoing process which can increase the perceived legitimacy of research findings. This review found only three studies that used semi-structured interviews to understand fisher–shark interactions more broadly, both of which included depredation. Gilman et al. (2008) took a global approach to understand and reveal trends in shark interactions with pelagic longline fisheries through interviews with fishers and port officials, finding that responses to shark depredation varied widely depending on whether sharks were viewed as nuisance bycatch or as byproduct. Key strategies that fishers reported using were moving location or switching bait types to avoid interactions (Gilman et al. 2008). Though not specifically targeting the shark depredation conflict, Iwane et al. (2021) conducted interviews with Hawaiian fishers to gauge and effectively define the problems within fisher–shark interactions, revealing that fishers viewed sharks as competitors for target species (through depredation), and more deeply, their livelihoods. Robinson et al. (2022) used semi-structured interviews to collect data on the impacts of shark depredation and how it affected fisher support for the Maldivian Shark Sanctuary established in 2010. Shark depredation was reported to cause high levels of catch and gear loss for reef fishers, although this was much lower for pelagic handline and pelagic pole and line fishers, with the latter even reporting that sharks sometimes play a beneficial role by pushing tuna up to the surface (Robinson et al. 2022). Most fishers (especially those who used to actively target sharks) had a negative view of the establishment of the shark sanctuary, reporting that it led to increases in shark populations and greater depredation (Robinson et al. 2022). As a result, 12% of fishers reported that they kill sharks as a means of reducing depredation, reducing the legitimacy and efficacy of the shark sanctuary (Robinson et al. 2022). These studies are consistent with surveys of recreational fishers in the southeast United States which found that “sharks threaten fishing efforts” (Drymon and Scyphers 2017). Likewise, Casselberry et al. (2022) found through surveys that fishing guides reported sharks to be a main threat to their livelihoods. Beyond these tangible problems, the interviews conducted by Iwane et al. (2021) also illuminated deeper fisher conflicts with management and science, which would likely have been missed through surveys alone. Gaining such valuable insights can help inform broader surveys to better quantify fisher experiences with shark depredation and provide guidance to managers and scientists about how to engage stakeholders successfully.

The limited survey and interview-based research conducted so far suggests that at a surface “dispute” level, the issue can derive from economic or physical losses and practical decisions like site or gear choices. Most of the current strategies being used to mitigate depredation (e.g. moving locations, decreasing soak times, modifying gear setups) are behavioural solutions (Gilman et al. 2008; Tixier et al. 2021), which primarily address this dispute level. However, underlying and deeply rooted conflicts around poor perceptions of management legitimacy and threatened fisher identities add complexity to the fisher–shark conflict, and call for broader interventions beyond these visible effects. Therefore, to effectively understand and address the complexities of shark depredation, these underlying conflicts and perceptions should be further explored and characterised.

#### Content analysis

Content analysis examines published material, such as magazine articles or social media content to explore the discourse surrounding a specific issue, event, or phenomenon. These analyses can range from simple enumeration exercises (e.g. how often a specific word is used), to more complex examinations of the themes, values, and meanings of the content. This methodology has been used to examine dominant narratives and attitudes in the media reporting on shark bites (Muter et al. 2013) and how attitudes towards sharks have changed over decadal time scales (Whatmough et al. 2011; Whitenack et al. 2021). While content analysis is a widely used social research tool, this review did not identify any examples applied to shark depredation. Nevertheless, content analysis could be very informative in understanding fisher experiences, perceptions, and attitudes towards sharks and shark depredation, and help guide scientists and managers in engaging with fishers on the issue. More specifically, content analysis could be used to identify common depredation experiences, perceptions regarding drivers of depredation and the potential solutions, and highlight conflicting narratives and associated values, beliefs, and attitudes between different groups. Therefore, content analysis could provide valuable opportunities for biologists to access fisher knowledge and could help managers in designing engagement activities. Nevertheless, content analyses need to be carefully designed and interpreted. Social scientists need to decide if sampling should be purposive or probabilistic based, to test keyword search strings for ‘recall’ and ‘precision’, and test for inter- and intra-coder reliability (Lacy et al. 2015). Furthermore, researchers may need to consider issues, such as ‘confirmation bias’ and ‘echo chambers’ in interpreting results where personal beliefs and values affect what content is created and how it is disseminated, as evidenced in social media induced polarisation regarding the COVID-19 pandemic (Modgil et al. 2021).

#### Participatory modelling

**Fig. 2:**
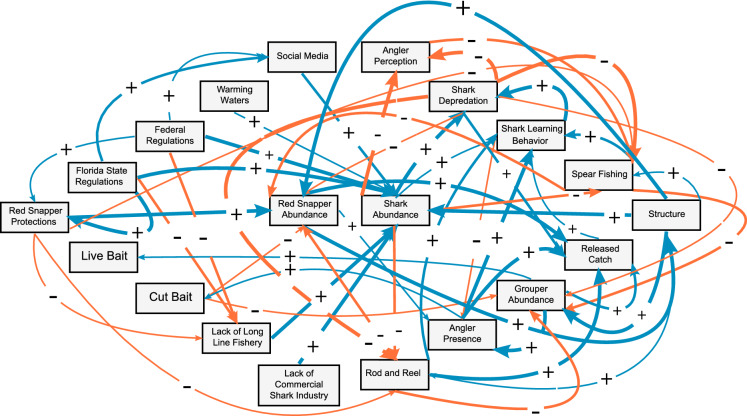
Cognitive map of a US offshore fisher’s mental model of depredation (Prasky et al. unpublished data), illustrating the complex and interconnected nature of depredation. Arrows indicate the direction and influence between concepts and can be positive (+), negative (−), or unknown (?). Arrow thickness indicates the strength of the effect

Participatory modelling techniques can also be used to study depredation. While no studies have yet applied these approaches to examine depredation, they are included here as a primer to guide future attempts. Among the most common participatory modelling approaches involves fuzzy-cognitive mapping (FCM) to represent individual or group “mental models” of a complex system, such as depredation (Fig. 2). Fuzzy-cognitive mapping has become increasingly popular in fisheries science to represent local ecological knowledge of complex issues, such as food web dynamics (Stier et al. 2017), climate change (McClenachan et al. 2020), and social-ecological interactions (Gray et al. 2020). In essence, FCM involves conducting interviews or focus groups to map out the most important components and causal relationships within a system. A major strength of FCM is the ability to develop models with (1) abstract (e.g. satisfaction) and aggregate (e.g. water quality) variables, (2) relationships that are not known with certainty, (3) feedback loops and cross linkages among model components, and often most importantly, (4) visual representations of scenarios or potential outcomes of policy options (Özesmi and Özesmi 2004; Gray et al. 2014). Compared to other social science approaches, FCM fits in a middle space between qualitative interviews, which are often easier to interpret but provide less detail on complex interactions or trade-offs, and more quantitative models that are often more challenging for stakeholders to interpret (Voinov et al. 2018).

### Biological data collection

Depredation is influenced by a range of factors, including shark behaviour and abundance, spatial and temporal distribution of fishing effort and changes in fishing methods, gear and equipment. However, there remains a lack of empirical data to characterise these issues, and consequently much of the current knowledge is composed of anecdotal accounts and personal opinions. Biological data collection can help to address key questions relating to depredation, such as the impacts of shark depredation on target species, which shark species are involved and how their movement patterns and behaviour influence the occurrence of depredation. Such information can then inform future work to design effective mitigation approaches.

#### Impacts on target species

Shark depredation can have substantial impacts on the target species of a diverse range of fisheries. In particular, shark depredation can increase the overall level of mortality that occurs, especially in fisheries where fishers are seeking to reach an allowed quota (commercial fishers) or bag limit (charter and recreational fishers). Depredation of target species in catch-and-release recreational fisheries causes mortality for released fish that would otherwise mostly survive (thus undermining the catch-and-release objective), as well as diminishing the recreational fishing experience and negating the benefits of tagging programs.

There are relatively few studies that have collected data on shark depredation rates for teleost target species (Table 2), predominantly in commercial longline fisheries where valuable tuna and billfish species are targeted. The results of these studies are detailed in IOTC (2007), Gilman et al. (2007) and Mitchell et al. (2018a). Carmody et al. (2021) also quantified shark depredation rates for narrow-barred Spanish mackerel (*Scomberomorus commerson*) in a commercial trolling fishery in Australia. Two studies in northwest Western Australia presented results for shark depredation rates for common recreational target species, including narrow-barred Spanish mackerel, spangled emperor (*Lethrinus nebulosus*) and coral trout (*Plectropomus spp*.) (Sumner et al. 2002; Williamson et al. 2006). Shark depredation impacting catch-and-release recreational flyfishing has been investigated with permit (*Trachinotus falcatus*) in the Florida Keys, USA (Holder et al. 2020) and bonefish (*Albula sp*.) in the Bahamas (Cooke and Phillip 2004) and French Polynesia (Lennox et al. 2017). Red snapper (*Lutjanus campechanus*) is another key target species known to be affected by shark depredation in the southeast USA (Streich et al. 2018; Drymon et al. 2019, 2020a).

**Table 2:** Research quantifying shark depredation impacts on target species/groups

Species/group	Gear type	Location	References
*Commercial fisheries*			
Tuna and billfish (*Makaira nigricans, Thunnus obesus, T. albacares*)	Pelagic longline	Pacific Ocean	Kobayashi and Yamaguchi (1978)
Swordfish and yellowfin tuna (*Xiphias gladius, T. albacares*)	Pelagic longline	Northwest Atlantic Ocean and Gulf of Mexico	Beerkircher et al. (2002), Mandelman et al. (2008), MacNeil et al. (2009)
Yellowfin tuna	Pelagic longline	British Indian Ocean Territory (BIOT), Indian Ocean	Clark et al. (2007)
Swordfish	Pelagic longline	Reunion Island, Indian Ocean	Poisson et al.(2007), Rabearisoa et al (2018)
Groundfish	Gillnet	Georges Bank, USA	Rafferty et al. (2012)
Spanish mackerel	Trolling lines	Western Australia	Carmody et al. (2021)
Swordfish	Harpoon	Strait of Messina, Mediterranean Sea	Malara et al. (2021)
*Recreational fisheries*			
Spanish mackerel and spangled emperor (*L. nebulosus*)	Hook and line	Northwest Western Australia	Sumner et al. (2002)
Coral trout (*Plectropomus spp.*)	Hook and line	Northwest Western Australia	Williamson et al. (2006)
Reef fish (e.g. Slinger (*Chrysoblephus puniceus*) and pelagic fish (e.g. yellowfin tuna, mahi mahi)	Hook and line	KwaZulu-Natal, South Africa	Labinjoh (2014)
Red snapper	Hook and line	Gulf of Mexico, USA	Streich et al. (2018), Drymon et al. (2019), Drymon et al. (2020a)
Permit	Hook and line	Florida Keys, USA	Holder et al. (2020)
Bonefish	Hook and line	Bahamas & French Polynesia	Cooke and Philipp (2004), Lennox et al. (2017)

Although focused mainly on depredation by toothed whales, NOAA Fisheries has developed standardised protocols to collect depredation rate data in an annual fishery-independent survey and account for depredation mortality when conducting stock assessments and setting quotas in the Alaska sablefish fishery (Hanselman et al. 2018). To allow the impacts of shark depredation to be incorporated into stock assessments, future data collection protocols should therefore be implemented to enable quantification of depredation rates at a whole fishery level, and for the main target species in multi-species fisheries, where possible.

#### Identifying shark species responsible for depredation

##### Video

Underwater video cameras have been used in several studies to investigate depredation (Mitchell et al. 2019, 2020; Van Den Hoff et al. 2017; Wiley and Pardee 2018; Coulson et al. 2022). This research has predominantly focused on specific fisheries to identify which species are depredating, the amount of catch lost, and the behaviour of depredating species. Research has been carried out under both experimental and observational settings, including controlled baited camera drops and camera deployment during operations on-board fishing vessels. Camera systems range from inexpensive action cameras to line mounted fishing cameras and expensive purpose-built deep-sea video camera setups, with all systems similarly limited in their effectiveness by weather, sea conditions, and high turbidity. These cameras varied in technological performance and capabilities, with differences in size, resolution, depth rating and camera shape.

Small action cameras (e.g. GoPro™) are commonly used to assess depredation. Video footage collected using GoPro™ cameras mounted to crab traps showed that the primary species responsible for depredation of spanner crabs (*Ranina ranina*) were species not often perceived as predators, such as those from the families Aetobatidae and Rhinidae, and that there are a variety of species that simply interact with bait rather than depredating catches (Wiley and Pardee 2018; Milburn 2021). Mitchell et al. (2020) also used GoPro™ cameras attached to a crossbar and suspended below a float to determine the time of arrival of sharks to a bait suspended below the cameras. In these two studies, the static nature of the crab trap or crossbar generated clear footage and enabled observation of the shark behaviour, interactions and identification of shark species. Drymon et al. (2020a) successfully used GoPro™ cameras to identify shark species depredating fish from descender devices (i.e., weighted return-to-depth tools which are used to improve post-release survival of captured teleosts, Bohaboy et al. 2020). In other research, GoPro™ action cameras have been found suitable for identifying sharks to species level, where sharks were inadvertently observed depredating other sharks and teleosts (O’Shea et al. 2015; Streich et al. 2018). The maximum depth rating of 50 m for the housing of GoPro™ action cameras is a limiting feature of these cameras, although aftermarket housings with deeper maximum depth ratings are available. Future development of underwater housings to extend the depth range and the use of red camera filters to further improve image quality could greatly improve the applicability of GoPro™ cameras in depredation research.

There are several commercially available underwater cameras that are designed to be attached to fishing lines (Table 3). These cameras provide the opportunity to collect important observational information before, during and after depredation events. Mitchell et al. (2019) used WaterWolf™ fishing cameras attached to the lines of charter fishing customers, two meters above the baited hook. While this enabled the behaviour and interaction of various shark and teleost species with bait and hooked fish to be observed and depredation rates to be quantified, the ability to identify the sharks to species level was relatively low (37%). Likewise, GoPro™ cameras on specifically designed fishing line camera mounts were able to capture clear footage of depredation events, but shark species identification was difficult (Coulson et al. 2022). In both cases, the inability to identify shark species is largely due to the chaotic nature of depredation events that cause the fishing line and camera to shake and spin rapidly as well as the subtle morphological differences between species, particularly species of the genus *Carcharhinus*. Other purpose-built, line-mounted fishing video cameras, such as Spydro™ and GoFish cam™, possess high image quality and LED lighting, which may address some of these issues, but not the difficulty in identifying shark species. In some situations, specialised underwater camera systems may be the only option. For example, specifically designed underwater camera systems outfitted with twin 500 lm lights, able to tolerate very low temperatures and depths of over 1000 m, revealed that southern elephant seals (*Mirounga leonina*) were depredating and interacting with hooked fish on a longline (Van Den Hoff et al. 2017).

**Table 3:** Examples of some underwater video cameras designed for use on fishing lines and a comparison of the features for each of those cameras

Product name	Cost	Max. Resolution	Camera angle (°)	Battery life (h)	Depth rating (m)	Other features	Comment
Water Wolf™	AU$199	1280p 30 FPS	120	4	100		No longer available
Spydro™	US$269	1080p 60FPS	130	3.5	150	Magnetic activation. Concealed unit	Requires a phone application to change camera settings and download recordings
GoFish™	US$150	1080p 60FPS		1.5	150		
Siren®	n/a	1080p 60FPS			100	Dual facing cameras, buoyant	Camera not available yet. Camera not intended to be attached to fishing line all the time, only attached after a fish is hooked
Hook Eye	US$399	1080p 30FPS	140	3	120	Magnetic activation	
GoPro 9/10	AU$669–819	5 k	123	2	60		Cameras may overheat in warm tropical waters. Water pressure > 40 m may cause camera to turn off. No fishing line mount commercially available

While video cameras have been used to successfully identify depredating species, their application in observing depredation behaviour and calculating depredation rates is still in its infancy. Given the variety of environments where depredation occurs, there is no single video camera system that can be used for all areas of research. When working in shallow depths, GoPro™ action cameras provide clear high-resolution video and allow potential identification of depredating species. When collecting data while line fishing on vessels using rod and reel, droplines or longlines, line mounted underwater cameras appear to be a good option. However, future research in line fisheries should consider trialling high resolution 360-degree underwater action cameras to collect high quality images of depredation. Further research should also trial line mounted fishing cameras while trolling to observe if depredation varies with different fishing methods. In addition, the use of underwater drones (ROVs, AUVs) to view depredation could also be trialled to see if the combination of greater manoeuvrability and a live camera feed is able to allow additional data to be collected.

##### Genetics

The use of genetic techniques for the identification of predator species responsible for depredation continues to grow, and shows increasing promise, with several research papers successfully using genetic approaches to identify depredating shark species from trace DNA collected off the remains of depredated species (Drymon et al. 2019; Fotedar et al. 2019; Vardon et al. 2021). This process is undertaken by swabbing the bite wound on the fish after depredation (Drymon et al. 2019; Fotedar et al. 2019). Although this method had been widely used across terrestrial environments (Williams et al. 2003; Blejwas et al. 2006; Caniglia et al. 2013; Fabbri et al. 2018), application in marine environments has only been recently undertaken (Drymon et al. 2019; Fotedar et al. 2019; Van Bleijswijk et al. 2014; Vardon et al. 2021). The application has now been trialled on commercial line (e.g. Webb et al. 2022) and net fisheries, as well as in charter and recreational fisheries, but considerable scope for broadening and enhancing the application of genetic approaches in depredation research exist.

Comparisons among shark depredation studies to date have revealed variability in successful genetic identification of depredating animals. The greatest success (100%) was achieved by Fotedar et al. (2019) using a combination of COPAN FLOQSwabs™ and a QIAGEN QIAamp™ Stool kit. Despite the notable success of these approaches, considerable opportunities remain to optimize the DNA collection, extracting and sequencing methods. Similar research by Drymon et al. (2019) reported a success rate of 61.5% using buccal swabs and the Omega BIO-TEK E-Z 96™ Tissue DNA Kit. Using trained fishers, Webb et al. (2022) reported 90% success rate. Although Fotedar et al. (2019), Drymon et al. (2019) and Webb et al. (2022) reported high success, sample sizes were low (16, 13, and 29, respectively). Research by Vardon et al. (2021) used a larger sample size of 52 but reported a lower success rate (19.2%), using polyurethane foam medium head swabs (Texwipe) and the QIAGEN DNeasy™ Blood and Tissue Kit. Unlike Fotedar et al. (2019) and Drymon et al. (2019), Vardon et al. (2021) used samples that had been collected and frozen by fishers before being defrosted and swabbed. Due to the varying methodologies, a comparative study using standardised testing would be required to determine which swabs and DNA extraction kits are optimal for use.

Variation in the time taken to swab depredated remains is also believed to affect the success rate of identifying depredating species. Vardon et al. (2021) found that depredating shark species can be identified from swabs taken from frozen depredated fish remains; however, high identification success was achieved by both Fotedar et al. (2019) and Drymon et al. (2019), which swabbed freshly depredated samples. The delay in swabbing depredated fish or freezing samples prior to swab sampling likely resulted in lower success as shark DNA would have degraded faster in situ on the fish than it would have if swabs had been collected immediately upon landing the depredated catch. Harms et al. (2015), recommended that swab samples be taken off depredated animals within 1–24 h. Additional research would benefit from investigating the amount of time after swabbing occurs before DNA degradation prevents the identification of depredating species (Drymon et al. 2019; Fotedar et al. 2019; Vardon et al. 2021). Variation also exists in the best way to swab a depredated sample to optimise depredating species identification. While research is limited, both Van Bleijswijk et al. (2014) and Wheat et al. (2016) found that swabs taken around teeth marks on depredated remains resulted in the most successful identification of depredating species. The optimisation of the collection and storage process presents a clear avenue for development where sample collection by non-researchers may be advantageous. If a standard protocol can be developed that allows for the collection of samples by non-researchers, then it would present enhanced capacity for ongoing monitoring of the issue and large-scale data collection through citizen science.

All genetic approaches to date have focused on traditional DNA sequencing methods, where primers are designed based on a presumed group of target taxa. In all instances so far, these are universal shark specific primers, which are designed to target several genera of sharks while not amplifying fish or human DNA. This approach is derived from traditional DNA barcoding applications that focus on conserved mitochondrial (mtDNA) regions, such as COI, ND2, ND4 and CtyB, which provide additional benefits when working with trace DNA due to the multi-copy nature of mtDNA. Several studies have also highlighted the importance of using primers that are specifically designed to amplify DNA of depredating species and block DNA of the species being depredated (Drymon et al. 2019; Fotedar et al. 2019; Van Bleijswijk et al. 2014; Vardon et al. 2021). Research by Drymon et al. (2019) and Fotedar et al. (2019) both used primers to target mitochondrial DNA from the gene region (cytochrome oxidase subunit 1; COI). Although often referred to as the barcoding gene, the COI gene has been found to be too conserved in sharks for the discrimination of closely related species (Ward et al. 2009). To overcome these challenges, Vardon et al. (2021) used primers to amplify the mitochondrial shark DNA NADH dehydrogenase two (ND2) and four (ND4) genes, which was successful in mostly identifying between different *Carcharhinus* spp., one of the most common genera responsible for depredation events studied to date (Mitchell et al. 2018a). Using both ND2 and ND4 improved species resolution. Thus, it is recommended that both ND2 and ND4 genes should be used in future research to maximise success in determining depredating species.

While traditional approaches using designed primers have been effective in selectively amplifying trace amounts of shark species DNA, this method is limited to the primer target group. Therefore, when the depredating species are not sharks, as has been noted in various studies (Gilman et al. 2008; Mitchell et al. 2018a, b, 2019; Rabearisoa et al. 2018), these single copy barcoding approaches become less effective. DNA metabarcoding presents a clear opportunity for improving methods to identify depredating animals by enabling simultaneous detection of multiple species by using a high-throughput sequencing platform. Metabarcoding approaches are now used routinely as part of environmental DNA (eDNA) studies and have been shown to be particularly effective in detecting the presence or absence of taxa to species level through water samples (Miya 2021). Their application in depredation studies has so far been limited to a single study that collected source DNA from fishing nets to investigate the depredation of a cetacean (De Bruyn et al. 2021). Yet, metabarcoding techniques could be challenging from a citizen-science point of view, since the risk of contamination is much higher and therefore maintaining sterility may not be feasible.

Depredation has been observed in net, longline, other hook and line, seine, trawl, and trap fisheries and as a result considerable opportunity remains for genetic identification in many of these unexplored fisheries. However, there are a unique set of considerations which may also need to be overcome. For example, the large phylogenetic distance among depredating predator and depredated prey species may increase the accuracy of identifying the predator at the species level (particularly where the predator and prey are closely related taxa). Furthermore, the ability to swab residual DNA of different tissue types (e.g. hard exoskeletons of crustaceans or molluscs) remains untested and could be either more or less suitable than swabbing of fish soft tissue. In some circumstances, examining the bite wound on a depredated fish or invertebrate could be used to distinguish between broader groups of depredating taxa (Gilman et al. 2007; IOTC 2007), if time and/or budget is limited for conducting genetic methods.

## Reducing shark depredation

The development of deterrent technologies that prevent interactions with humans (bites) (Huveneers et al. 2018) or reduce shark bycatch while not decreasing catch rates of target species (Robbins et al. 2011), provides useful insights into potential technical applications to reduce shark depredation. Past studies have tested deterrents using static baits; however, it must be considered that the stimulus for a shark to depredate a struggling hooked fish is likely to be stronger than feeding on static bait, so the potential effectiveness of deterrents for reducing depredation may be lower than when static bait is used. Various types of shark deterrents have been investigated including magnets (O’Connell et al. 2014a, b), electropositive lanthanide metals (Brill et al. 2009; Kaimmer and Stoner 2008; Robbins et al. 2011), electrical (Howard et al. 2018; Verschueren et al. 2019), acoustic (Chapuis et al. 2019), and chemical (Stroud et al. 2014; Broadhurst and Tolhurst 2021) deterrents. There has also been significant work investigating the effectiveness of personal electrical shark deterrents for ocean users, such as surfers and divers (Gauthier et al. 2020; Huveneers et al. 2018; Marcotte and Lowe 2008; Thiele et al. 2020). Only recently has this technology been adapted in the development of deterrents for use in recreational fisheries, with a single study investigating their effectiveness. While the probability of depredation was not significantly reduced during fishing sessions when the use of three types of deterrent [magnetic (“SharkBanz Fishing—Zeppelin” n.d.), electrical (Ocean Guardian 2019) and acoustic (“SharkStopper” n.d.)], those deterrents were, collectively, effective in reducing the overall proportion of fish depredated by sharks (by more than 60%) and increased the time taken for fish to be depredated after becoming hooked (Department of Primary Industries & Regional Development [DPIRD], Western Australia, unpublished data).

Magnetic, electropositive lanthanide metal and electrical deterrents all work on the same premise of overwhelming the electrosensory system of the shark and thus evoking avoidance behaviours. The electrosensory system has been demonstrated to override other sensory modalities, with sharks documented to ignore the visual and chemical stimuli produced by nearby food items to preferentially bite at electrodes (Kalmijn 1972; Kajiura 2003). Therefore, a mitigation strategy that targets the electrosensory system may provide a mechanism to selectively deter sharks from biting while not affecting teleost fishes. The use of lanthanide metals as shark deterrents has been investigated with mixed results. Some studies demonstrated shark avoidance to the metals, while others did not (reviewed in McCutcheon and Kajiura 2013; O’Connell et al. 2014a, b). There are significant limitations to the lanthanide metals which makes them unsuitable for recreational or commercial application. These metals are expensive and are hazardous to machine (Smith 2013). They are also classified as potentially toxic and should not be stored in air or in moist environments, so are unsuitable for use on a fishing vessel. Finally, they dissolve in water which would necessitate frequent and thus costly replacement (McCutcheon and Kajiura 2013).

Magnets have been investigated as a potential alternative to lanthanide metals. A shark swimming near a magnet will induce an electric field around its body that is potentially detectable by its electrosensory system (Kalmijn 1974). Various types of permanent magnets, including ferrite (Fe_2_O_3_), barium ferrite (BaFe_12_O_19_), and neodymium ferrite (NdFeB) have had varied success as shark deterrents and are not always effective, particularly when there is competition among sharks during feeding (Robbins et al. 2011; DPIRD unpublished data). Moreover, the effective range of many magnets is very small, requiring a shark to be in close proximity before being deterred (O’Connell et al. 2014a, b; Rigg et al. 2009). Recent work mapping the magnetic field intensity around a commercially available deterrent (“SharkBanz Fishing—Zeppelin”) illustrates that the magnetic field decreases to background levels approximately 30–40 cm from the device (S. Kajiura, unpublished data), largely because of the physical properties of the magnetic field, which decays with distance as an inverse square function (Kalmijn 1974). This produces a limited effective range which necessitates that the device be positioned close to the hooked fish to provide adequate protection (S. Kajiura, unpublished data).

Electrical deterrent devices work by using a battery to drive an electric current between pairs of electrodes, which produces an electric field around the device. Previous studies investigating the effectiveness of personal shark deterrents determined that the Shark Shield Pty Ltd (Ocean Guardian 2019) devices are consistently more effective than other deterrents (Huveneers et al. 2013, 2018). This technology has been applied to create the Shark Shield Pty Ltd Fish01 device which is intended to deter sharks from an area 3 m either side of the device (Ocean Guardian 2019). The Fish01 device was effective in deterring sharks when hooked fish were within the effective range of the device but is limited because the position of the device in the water column is fixed and potentially too far away from the fishing line (DPIRD unpublished data). There is also a risk that fishing lines would get tangled around this device, which may limit its practicality for use on larger vessels where multiple lines are in the water at the same time. Smaller devices that could be deployed directly on recreational fishing gear have been demonstrated to significantly reduce bait consumption (Howard et al. 2018). However, the system would need significant refinement to create a commercially viable product that is applicable for reducing shark depredation in a fishing setting.

The use of acoustics as a shark deterrent has been investigated, particularly the use of orca (*Orcinus orca*) calls. While these have been shown to be an effective shark deterrent (Chapuis et al. 2019; Myrberg et al. 1978), sharks also exhibit evasive behaviours when exposed to artificially generated sounds that are rapidly increased or suddenly transmitted, even at a low amplitude (Banner 1972; Collin 2012; Hart and Collin 2015; Myrberg 2001). However, an important consideration in the application of acoustic deterrents is that sharks become habituated to attractive and repulsive noise (Myrberg et al. 1978, 1969), as well as the impact that transmitted sounds may have on other marine fauna, such as cetaceans and target fish species (Wartzok et al. 2003).

Another proposed avenue for reducing depredation is to employ shark-specific necromones. Sharks have been reported to be deterred by the odour of necromones from decaying shark tissue and have avoided the area for up to 10 min (Stroud et al. 2014). Applying necromone dispersing canisters to fishing gear might deter sharks from the gear. However, the impact on target teleost fishes would need to be more thoroughly explored. One study found that teleost fishes remained in the presence of a shark necromone whereas two shark species were deterred, which suggests that the response was specific to sharks (Stroud et al. 2014). Yet, in another study, the authors found that the odour of shark flesh from a mixture of species produced no reduction in shark catch on longlines (Broadhurst and Tolhurst 2021), which challenges their utility. Responses of sharks to necromones are likely to be complex and possibly species-specific, so further research is required to increase understanding of whether they can be viable as deterrents.

### Protocols for testing deterrent devices

The development of shark deterrent devices is still in its infancy with several companies developing deterrents for this purpose. It is therefore inevitable that there will be a need to undertake independent testing of these devices. Establishing scientific evidence that deterrents are successful can be difficult and time consuming, yet critically important for independently validating product claims from device manufacturers. Recent methods to test the efficacy of personal shark-bite deterrents for surfers have been established (e.g. Huveneers et al. 2018); similarly, it would be advantageous to develop a set of protocols that could act as a guide in testing the effectiveness of current and future shark depredation deterrent devices.

When determining the effectiveness of shark deterrent devices, the sampling design should consider the appropriate sample size required to detect whether deterrents are effective in reducing the probability of shark depredation by determining (1) the level of statistical power required, (2) the current base-level of shark depredation (i.e., without shark deterrents), (3) the level of decrease in depredation that is required for a deterrent to be considered “effective”, and (4) the change in effectiveness over time since the initial exposure (i.e. whether habituation occurs in the sharks). When planning and carrying out field sampling it is necessary to consider: (1) proximity of sampling sites to one another, (2) adequate numbers of catchable fish and sharks are present, (3) duration of fishing session to enable fish to be caught and sharks to be encountered, (4) randomisation of the order in which a control and each deterrent device are tested while fishing, (5) maintaining the same number of fishing lines in the water throughout the sampling, and (6) maintaining consistency in the fishing equipment/hardware (i.e., number of hooks, hook size, line class) used throughout the sampling (Table 4). Yet despite best efforts, some variables are beyond control (e.g. the size and species of fish hooked), exemplifying the inherent challenges with attempting manipulative experiments in a field setting.

**Table 4:** Variables to be considered when field testing the effectiveness of shark deterrents for reducing shark depredation

Variable	Consideration
Sampling region	Sufficient numbers of fishing sites to (1) deploy each deterrent and the control multiple times and (2) avoid fishing sites more than once. Allow for sufficient distance between sites (1–5 km)
Fishing sites	Sufficient numbers of fish present that can be caught consistently during the fishing session. Sufficient number of sharks present
Length of fishing sessions	Ensure enough time to catch fish and interact with sharks (if present)
Deterrent device/control	Randomise the order of use
Number of fishers	Greater numbers of fishers may lead to stronger attractant cues for sharks. 3–6 fishers replicate recreational scenarios, while 6 + fishers replicate charter scenarios
Fishing hardware	Fishing line class, number of hooks and fishing method remains consistent
Fish species hooked	Sharks may be more or less likely to depredate certain species of fish, as documented by Mitchell et al. (2019). Some shark species may be more likely to depredate
Shark species present	Some shark species may be more aggressive and likely to depredate hooked fish than others. Interspecific interactions, such as competition and dominance hierarchies may influence the likelihood of a shark deterrent being effective, as documented by Robbins et al. (2011) and O’Shea et al. (2015)

### Future testing

Most research in the depredation field has focused on the loss of fish that are hooked and being reeled in by the angler. However, some fish are returned to the water while attached to fishing gear (e.g. descender devices) and are thus still subject to depredation. The only study that has investigated this aspect indicated that fish are far less likely to be depredated on descender devices (Drymon et al. 2020a), but note these findings are likely geographically variable. In addition, without directly seeing what animal is depredating a fish, most fishers will suspect that sharks are responsible. However, some teleost species that grow to large sizes (> 1 m), i.e., *Epinephelus malabaricus*, *E. tukula*, *E. itajara*, *Hyporthodus nigritus*, *Sphyraena barracuda*, *Seriola dumerili*, have also been reported to depredate teleosts (Streich et al. 2018; Shideler et al. 2015; DPIRD unpublished data) and may not be affected by deterrent devices aimed to mitigate shark depredation. Understanding what proportion of catch is depredated by teleosts will be an important consideration, particularly in those regions where some of these species are protected, such as goliath grouper (*Epinephelus itajara*) in Florida (Shideler et al. 2015).

## Managing shark depredation

The sections above provide a review of the methods and results of research used to characterise and mitigate shark depredation. An improved understanding of this issue is critical for fishery managers to develop suitable policy responses and respond to stakeholder concerns. To date, we are not aware of any specific management responses to address shark depredation. Despite this, there are several courses of action available to managers and fishers. While the most commonly suggested mitigation approaches are technical (i.e., shark deterrent devices, see “Reducing shark depredation” section) and geared towards reducing or eliminating the physical act of depredation, additional approaches for managing depredation exist, including reducing shark numbers, changing fisher behaviours, and educating stakeholders. We explore the limitations and challenges of these approaches below.

### Reducing shark numbers

One solution to depredation advocated by some fishers is to reduce shark numbers by opening or increasing commercial fishing for sharks or even culling (Kagi 2016). Both approaches assume that depredation rates are proportionally related to shark abundance rather than changes in shark behaviour. If this is true, then to achieve appreciable reductions in shark depredation it would be necessary to reduce shark populations to very low levels compared to current levels, which is not allowed in countries with legal mandates to end overfishing (e.g. US and Australia). In many situations, fishers have claimed that increasing shark populations following the protection or management of sharks are responsible for increasing depredation rates. The assumption about a relationship between shark abundance and shark depredation rates remains to be tested, but underscores why fishery managers require scientific studies to underpin decision-making since it can have significant implications for shark populations (some of which are already depleted and threatened with extinction) and the wider marine ecosystem (Heithaus et al. 2008). Unlike other solutions, this approach would require significant involvement from fishery managers and would likely require some form of regulation (e.g. to allow sharks to be caught and killed).

There are many challenges to this type of approach. Firstly, knowing which species of sharks are responsible for depredation would be necessary to focus fishing efforts on these species. However, even if the species are known, selectively fishing for these species is difficult given the low level of species-selectivity normally achieved in shark fisheries (e.g. Smart et al. 2020), so species not responsible for depredation may be unnecessarily depleted. Secondly, sharks identified as depredating species may currently be subject to fishing and thus managed already. In cases where management is maintaining a population at a sustainable level, added catches would affect sustainability and the long-term economic value of the fishery. Thirdly, some shark species are already overfished and of conservation concern because of their elevated extinction risk (Dulvy et al. 2014). Fourthly, for shark species where there is no information on population status or modelling to predict the consequences of increased catches, the challenges for managers are even greater. Finally, managing shark catch to reduce depredation would have a cost to the management agencies involved. There are also other costs to the ecosystem through the loss of important sources of predation that could result in unintended changes in these systems.

An example from Western Australia provides a useful case study to explore the feasibility of reducing shark numbers as a management tactic for shark depredation. There, the dusky shark (*Carcharhinus obscurus*), a known depredating species (Fotedar et al. 2019), is already subject to managed levels of commercial fishing (Braccini et al. 2018) and has a life history that makes it susceptible to overfishing, including late onset maturity (> 20 years), relatively low fecundity (2–18 pups) and infrequent reproductive periodicity (2–3 year reproductive cycle) (Simpfendorfer et al. 2002; Dudley et al. 2005; Natanson et al. 2014). While the dusky shark is only one of the shark species identified to depredate fish in this region (Fotedar et al. 2019), increasing shark fishing to reduce depredation would likely lead to overfishing of dusky sharks that are still recovering from historic overfishing after years of careful management (Woodhams et al. 2021). Reducing dusky shark abundance at local scales where depredation is an issue is also not viable because the population moves over large distances (> 1000 km) along the Western Australian coast (Braccini et al. 2017; Bartes et al. 2021). To communicate these concepts, a fact sheet on shark depredation in Western Australia was developed to share details on the history of commercial shark fishing, current status of the key shark stocks, and results from recent depredation research, to educate fishers and potentially encourage changes in their fishing behaviour (Anon 2021).

For managers considering reopening commercial shark fisheries to reduce shark populations and shark depredation, there are also important considerations around marketability of the sharks caught, as there is typically low value for shark meat (Ferretti et al. 2020) and large sharks have high concentrations of mercury and other contaminants (polychlorinated biphenyls, PCBs) in their flesh (Pethybridge et al. 2010; Gilbert et al. 2015; Tiktak et al. 2020). Overall, the practicalities and costs of reopening and managing commercial shark fisheries at a sustainable level to reduce depredation are complex. Many of these complexities are not apparent to all stakeholders; therefore, it is important that efforts are made to provide clear messaging around the history of management of shark stocks and limitations of reopening commercial shark fisheries or culling sharks to reduce shark depredation in many jurisdictions where the wider public support (‘social license’) for such approaches does not exist.

Reducing shark populations to address depredation issues poses such significant challenges to fishery managers that they are unlikely to be widely used. However, this may be unpopular with some stakeholders who consider this as the only solution. The danger in that situation is that fishers take matters into their own hands and start killing sharks (Carlson et al. 2019; Casselberry et al. 2022), which would have the potential to reverse positive trends in shark conservation. While pressure from fishers to increase the catch of (or even cull) sharks can be significant, there is likely to be equally strong pressure from conservation groups not to increase shark catches given their conservation status (Dulvy et al. 2014; Pacoureau et al. 2021). This is where the learnings from terrestrial HWC can be used to help design strategies to help address the needs of all stakeholders in the debate and reduce the human–human conflict that arises from different values and beliefs about sharks (Simpfendorfer et al. 2021).

### Changing fisher behaviour

Reducing shark depredation via changes to fisher behaviour aligns closely with the technological approach. This requires that fisher behaviours that effectively reduce shark depredation can be identified via appropriately structured research. Managers may play a role in advocating for the adoption of behaviours once research is available, but top-down regulation is unlikely. Unlike technological solutions, behavioural change would not have a direct economic cost, but may have indirect economic costs (e.g. if changes in fishing location were required more often than previously, increasing fuel costs). Behaviour change may also result in indirect-economic costs, such as reduced enjoyment and reduced catches.

Fishers have reported testing a wide range of modifications to fishing methods to try and mitigate depredation (Mitchell et al. 2021; Coulson et al. 2022). These can be related to the fishing location, including rotating fishing areas in a systematic way so as not to visit the same location too frequently, which will reduce the chances of sharks associating vessels with food at that location, or moving on from a given fishing location after a short time (e.g. after a few fish have been caught successfully or once sharks are sighted). Identifying locations where sharks may be less likely to occur, such as certain depth ranges, can also help to manage shark depredation (Mitchell et al. 2021). Fishing methods can also be modified to retrieve hooked fish more quickly (i.e., using electric reels or heavy class line), or by using jigs and lures instead of bait to reduce odour cues and reducing or eliminating fish waste discards can also help to prevent sharks from associating fishing vessels with an easily accessible source of food (Mitchell et al. 2021; Coulson et al. 2022). In some areas, diversifying target species may help to mitigate shark depredation because fishers have anecdotally reported that sharks can be more likely to depredate certain fish species, such as tuna (*Thunnus spp.*) and yellowtail kingfish (*Seriola lalandi*), compared to demersal species (Mitchell et al. 2021). However, the reasons behind these apparent preferences of sharks are not yet well understood so would need to be investigated and quantified with further research. Additionally, the success of this approach would depend on the shark species present in the area and the fishing methods used. To increase understanding of potential modifications to fisher behaviour and to identify which methods have been successful, survey approaches such as those described previously could be useful. For example, in their review of marine predator depredation, Tixier et al. (2021) found that behavioural responses and gear modifications were the most effective strategies for reducing depredation, while Coulson et al. (2022) found that charter and recreational fishers simply move spots or stop fishing to minimise shark interactions. Gilman et al. (2007) found that altering longline soak times and hook depth are strategies that fishers have used to reduce depredation in some circumstances. Rather than modify gear or move locations, Casselberry et al. (2022) noted that recreational fishers who experienced depredation in the United States were more likely to target and harvest sharks. Mitchell et al. (2021) conducted a survey of recreational fishers at Lord Howe Island, Australia, to collect information on fisher practices, which was then converted into a list of best practice guidelines for fishers to refer to, particularly for visiting recreational fishers who had limited experience of the local fishery. Information on best practice fishing methods can be communicated to fishers using targeted education approaches, to ensure wider uptake. This approach could be particularly beneficial to recreational fishers who have not experienced shark depredation in the past.

### Engaging and educating stakeholders

Engaging and educating fishers and other stakeholders can provide deeper insights into the shark depredation conflict and help direct more innovative management options. Finding suitable compromises that account for societal conservation values and potentially conflicting fisher perspectives will likely prove challenging for managing shark depredation, akin to other human–shark conflicts (Gibbs and Warren 2015). Thus, working to uncover common values between stakeholders may help guide research and management strategies that align with multiple stakeholder priorities. Surveys have shown promise for quantifying depredation and gauging fisher attitudes (e.g. Drymon and Scyphers 2017; Ryan et al. 2019) and potential responses (e.g. Casselberry et al. 2022; Coulson et al. 2022) towards sharks. Such information can help reveal the extent that depredation may impact fisheries to further inform stock assessments and fisheries management. An additional role for social science research could be in identifying and communicating areas and times of high shark depredation so that fishers can avoid these areas and times (e.g. Carmody et al. 2021; Mitchell et al. 2018a, b). For example, findings from Holder et al. (2020) prompted a stakeholder driven movement to enact a time-area closure in the Florida Keys to protect spawning permit aggregations from depredation.

Engaging fishers more effectively in shark depredation research is key to better understanding the extent of the conflict, but effectively communicating research findings back to fishers is also crucial. While studies have shown that research institutions can be viewed as more trustworthy information sources (MacKeracher et al. 2018), fisher distrust of scientists has been recognised around the world, and often stems from communication barriers between stakeholders and scientists (Dedual et al. 2013). However, distrust can also form from unmet expectations (Hartley and Robertson 2008), lack of transparency throughout the research process (Iwane et al. 2021), or inconsistencies between fisher experiences and scientific results (Chambers and Carothers 2017). A more prominent role for science communicators may assist in overcoming such obstacles relative to depredation. Li et al. (2010) conducted a fisheries communication study in Queensland, Australia and found that recreational fishers had an acute interest in fisheries science, so more effective science communication could help bridge this gap. Studies have also shown that fishers use a range of information sources (e.g. websites, conventional media, online forums, social networks), and that the penetration of information and trust in the messaging can vary widely between these sources (Li et al. 2016). As such, effective communication and expectation management may vary between different communities and fisheries groups, and researchers and managers need to identify their specific audiences and the best means to effectively communicate and engage with them. Effective engagement of fishers throughout the research process can also be a powerful means to build trust in the process and the legitimacy of research findings. Mease et al. (2018) provide extensive guidance on effective stakeholder engagement to build trust and manage expectations including starting early, communicating often, being inclusive and transparent, and humanising management and managers. Taking the effort to deliver well planned, timely, and meaningful engagement throughout the research process is widely recognised in fisheries management, with numerous case studies that provide guidance on effective engagement processes and techniques (e.g. Iwane et al. 2021).

A commonality within the current body of research that has investigated the effectiveness of depredation mitigation approaches is that none are a “silver bullet” for preventing depredation. The currently limited availability of affordable and effective shark deterrent devices for fishing will require fishers to be willing to consistently modify their behaviour to adapt to changing fishing conditions to mitigate depredation. While this is already occurring in some regions (e.g. Lennox et al. 2017), in most areas where depredation is prevalent, additional work is required to encourage fishers to be proactive in their approach to mitigation while fishing (Janc et al. 2021). For fishers to make informed changes to their fishing behaviours to minimise shark depredation, it will require effective communication between scientists, policy makers and stakeholders (Dedual et al. 2013; Fairclough et al. 2014; Runde 2019).

## Discussion

Despite recent interest and advances in shark depredation research, we are still at a relatively early stage of characterising the extent of depredation and its impacts on fisheries, the shark species involved, identifying technical methods for deterring depredation, and managing depredation. This review has highlighted some of the recent progress made in understanding and quantifying depredation, particularly in the United States and Australia, as well as identifying future research gaps to be addressed. As part of this process, it is vital to understand how shark depredation fits into the broader context of fisheries management and shark conservation.

The issue represents an extremely challenging space for fishery managers, decision-makers, and politicians. Many stakeholders believe shark depredation is increasing due to increasing shark populations, particularly after the closure of some shark fisheries in the US and Australia. However, there is minimal long-term scientific data to investigate changes in abundance of sharks and there is no clear evidence to link changes in relative abundance of shark to increases in depredation rates. Studies from Australia (Braccini et al. 2020) and the US (Peterson et al. 2017) have shown relatively stable trends in shark populations over the last 10–15 years with only modest recovery in some species such as sandbar shark (*Carcharhinus plumbeus*). Yet, there are widespread anecdotal reports that shark populations have increased as fishers report seeing many more sharks around their vessels than in the past. While it is possible that some local populations of sharks have increased, particularly species with more productive life history traits and relatively high site fidelity (Pardo et al. 2016), it is also possible that reports of ‘greater’ shark abundances reflect a shifting baseline (sensu Pauly 1995). Dusky sharks in Western Australia and sandbar sharks in the US are two populations that exemplify this concept. Both stocks were historically overfished yet are now rebuilding under current management measures (Braccini et al. 2020; Peterson et al. 2017). Although fishers may be experiencing recent increases for these two species, populations are likely still lower than before they were overfished. Surveys offer a promising tool for documenting these generational changes in fishers’ perceptions of historic and modern shark populations (e.g. Powers et al. 2013). Further confounding these population-level trends is a need to better understand how shark species distributions are responding to climate change, such as potential range extensions of tropical species into areas where they may not have historically been observed (Last et al. 2011; Bartes and Braccini 2021).

It is also important to disentangle reported changes in relative abundance from the potential influence of changes in shark behaviour when investigating reports of increased depredation. In some areas there has been an increase in recreational fishing activity, such as in remote parts of Western Australia in recent years due to the COVID-19 pandemic (Ryan et al. 2021), so this increasing presence of vessels may have driven habituation of previously naïve sharks and development of associative behaviours where they associate the sounds of boat engines with the availability of food in the form of hooked and/or released fish. This scenario has also been suggested for sandbar sharks in areas of the northern Gulf of Mexico (Drymon et al. 2020b). In these instances, there would likely be a trend where fishers encounter more sharks and therefore perceive that their populations have increased. Changes in fishing practices may also exacerbate these trends. For example, in some areas where commercial trawling used to occur but has now been drastically reduced due to management measures, sharks that used to follow trawlers and feed on discarded fish would no longer have access to this food source, so may switch to following recreational fishing vessels instead.

It is necessary to consider how to manage stakeholders impacted by shark depredation scenarios, as well as the sharks. In several other HWC scenarios, the focus has shifted from one focused solely on the predator responsible, such as where the predominant management approach is to cull or catch and relocate the predator (Reynolds and Tapper 1996), to one focused more on managing human behaviour and the impacts on stakeholders, e.g. by subsidising changes in behaviour that support coexistence with predators (Ravenelle and Nyhus 2017). From a shark depredation context, a key process will be to learn more about how fishing methods can be modified to reduce the occurrence of shark depredation and then communicate this information effectively to fishers. Indeed, since fishing began, fishers have constantly experimented and adapted their fishing techniques to achieve higher catch rates, such as by testing different bait and lure types, targeting different phases of the lunar and tidal cycles and developing new technology, such as echosounders and electric reels. Therefore, modifying fishing methods to overcome the challenge presented by shark depredation is another scenario where adaptation can generate improvements in fishing.

Regardless of the methods used to mitigate depredation, it is highly unlikely that this HWC will be eliminated completely (Lennox et al. 2018). Indeed, if efforts to rebuild historically depleted shark populations are successful, depredation may potentially increase (Carlson et al. 2019). Moving forward, it will be necessary to set realistic goals and manage expectations towards reducing depredation to a tolerable level. Shark depredation sits within the context of a range of other human–shark interactions (Simpfendorfer et al. 2021). The range of functional roles of sharks and the multidimensional aspects of their conflicts with humans (ecological, social, economic) make mitigation of conflict difficult since solving one issue may introduce additional unintended consequences related to another level or type of conflict.

In the future, we can apply lessons learned from similarly contentious HWCs in the terrestrial realm. Recent meta-analyses indicate that reactionary predator removal programs in terrestrial ecosystems are rarely successful in reducing livestock depredation by apex predators (Eklund et al. 2017; Bruns et al. 2020) and in some cases, have even increased depredation (Treves et al. 2016; Smith and Appleby 2018). Efforts to continue to characterise shark depredation should lean on these insights as we move toward developing effective mitigation and management strategies. Ultimately, given the polarising nature of shark depredation, developing effective messaging and education for the diverse range of stakeholders affected is critical for minimising future conflicts.
